# Laser-Induced Evoked Potentials in the Brain after Nonperceptible Optical Stimulation at the Neiguan Acupoint: A Preliminary Report

**DOI:** 10.1155/2012/292475

**Published:** 2012-04-17

**Authors:** Gerhard Litscher, Guenther Bauernfeind, Gernot Mueller-Putz, Christa Neuper

**Affiliations:** ^1^Stronach Research Unit for Complementary and Integrative Laser Medicine, Research Unit of Biomedical Engineering in Anesthesia and Intensive Care Medicine, and TCM Research Center Graz, Medical University of Graz, Auenbruggerplatz 29, 8036 Graz, Austria; ^2^Institute of Knowledge Discovery, Graz University of Technology, 8010 Graz, Austria; ^3^Department of Psychology, Neuropsychology, Karl-Franzens University of Graz, 8010 Graz, Austria

## Abstract

We report on small but reproducible human cerebral evoked potentials after bilateral nonperceptible laser needle (658 nm, 40 mW, 500 **μ**m, 1 Hz) irradiation of the Neiguan acupoint (PC6). The results which are unique in scientific literature were obtained in a 26-year-old female healthy volunteer within a joint study between the Medical University of Graz, the Karl-Franzens University of Graz, and the Graz University of Technology. The findings of the 32-channel evoked potential analysis indicate that exposure to laser needle stimulation with a frequency of 1 Hz can modulate the ascending reticular activating system. Further studies are absolutely necessary to confirm or refute the preliminary findings.

## 1. Introduction

The irradiation of the skin overlying the median nerve of the wrist in humans using a helium-neon laser stimulation with a wavelength of 632.5 nm, an output power of 1 mW, and a frequency of 3.1 Hz, can produce a somatosensory evoked potential obtained at the Erb's point on the shoulder. This evidence of photosensitivity in peripheral nerves was found by Walker and Akhanjee already in 1984 [1].

The laser needle technology was invented at the University of Paderborn and first investigated scientifically at the Medical University of Graz [2]. The method does not puncture the skin; the needles are only applied at the surface of the skin.

The aim of this preliminary paper was to investigate if there are any measurable evoked potentials in the brain after “laser needle” stimulation at the Neiguan acupoint (PC6), which is also located at the wrist, near the median nerve.

## 2. Materials and Methods

### 2.1. Subject

**Figure 1:**
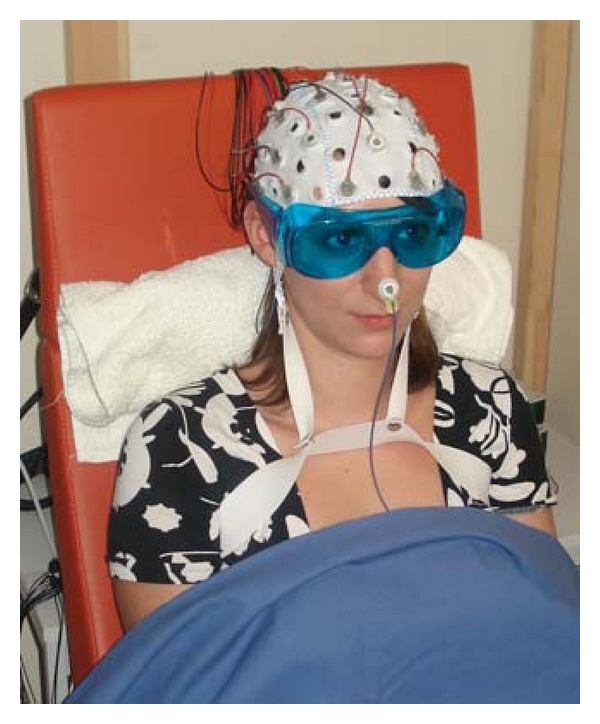
Healthy volunteer during “laser needle” EEG (electroencephalography) experiment in Graz, Austria (with written permission of the subject).

The subject for our investigations was a 26-year-old female who was sitting in a special sound booth. The box is located at the Department of Psychology, Neurophysiology (Figure 1).

Written informed consent was obtained, and the investigations were approved by the ethics committee of the Medical University of Graz (13-048, laser needle stimulation). The subject was not taking medications and had no neurological or psychological impairments. The volunteer was informed about the nature of the investigation, as far as the study design allowed, and the measurements were performed in accordance with the Declaration of Helsinki.

### 2.2. Laser Needle Stimulation

Laser needle stimulation (Laserneedle GmbH, Berlin, Germany) allows the continuous stimulation of one or more acupuncture points on the body, the head, hands, or ears [2–14]. In this investigation, laser irradiation of 658 nm and 55 mW laser diodes was coupled into an optical fiber, and the laser needle was arranged at the distal end of this fiber. Due to coupling losses, the output power of the laser needles was reduced to 40 mW. The fiber core used in this study was about 500 *μ*m in diameter. Stimulation frequency was 1 Hz. The method is described in detail in previous publications [2–14]. 

**Figure 2:**
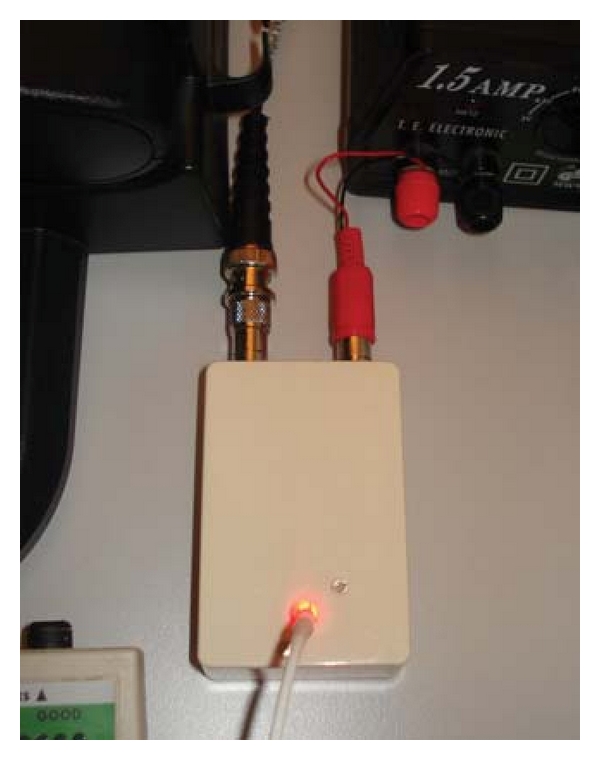
Trigger realization, especially for this laser needle study.

In order to have the laser pulses act as the sole stimulus to the average with the EEG (electroencephalography) recording system, each flash of the laser was accompanied by a 5 V pulse signal that triggered the average (Figure 2).

As in further studies [1], control experiments were performed to eliminate the possibility that the evoked potential obtained after laser irradiation was influenced by timed artifacts arising from the interface between the laser instrument and the signal averaging system.

### 2.3. EEG Data Acquisition and Analysis

For the EEG investigations we used two USB biosignal amplifiers (g.USBamp generation 3.0) with 16 input channels each. For all channels, the simultaneous sample rate was set to 512 Hz (24-bit resolution) with a high-pass filter at 0.1 Hz, a low-pass filter at 100 Hz, and a notch filter at 50 Hz [15].

In the present investigation, we recorded 32 EEG channels. The electrodes were positioned on the cap according to the 10-20 system; the reference electrode was placed on the nose, and the grounding electrode was placed behind the ear above the mastoid process (compare Figure 1). Electrode impedance was less than 5 kOhm in each position [15].

The software package MATLAB was used for the analysis of the evoked potentials. After a visual inspection of the raw EEG data, trials containing artifacts were marked and omitted from further analysis. Afterwards the raw EEG was band pass filtered between 0.8 and 10 Hz. Finally, altogether about 600 trials were averaged from the processed EEG.

### 2.4. Control Measurement

In the control measurement, the optical stimulation of the laserneedle was visible for the healthy volunteer. To avoid a direct stimulation of the eye, the subject wore eye protection glasses, and the light source was located behind a screen.

### 2.5. Optical Acupuncture and Placebo Stimulation

**Figure 3:**
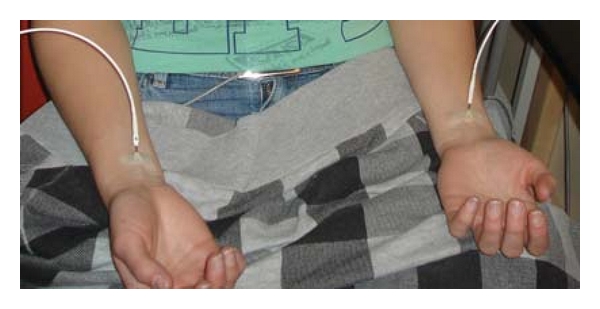
Bilateral stimulation of the Neiguan acupoint (PC6).

The laser needles were placed on the skin at the Neiguan (PC6) acupuncture point bilaterally (Figure 3). PC6 is situated between the tendons of the palmaris longus and flexor carpi radialis muscles, 2 cun proximal to the transverse crease of the wrist [16, 17]. The stimulation with red light (658 nm) was not felt by the subject. The volunteer has open eyes; however, the stimulation area was covered, and therefore the subject could not see whether the stimulation was on or off (compare Figure 1).

## 3. Results

**Figure 4:**
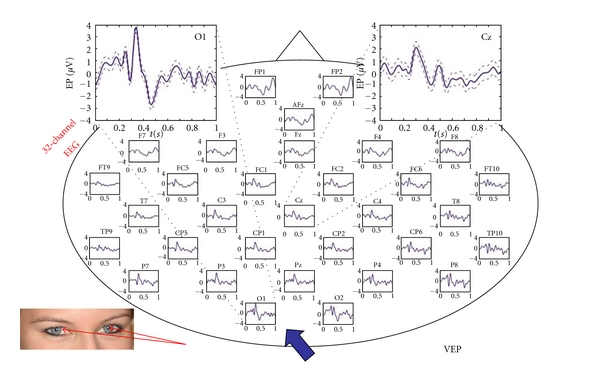
32 visual evoked potentials after visual input (indirect stimulation with laser light of 658 nm). Note the VEPs are present over the entire scalp with a dominance over the occipital (blue arrow) and central regions (*X*-axis: s; *Y*-axis: *μ*V; mean ± SE).

The result of the 32-channel visual evoked potential analysis is displayed in Figure 4.

**Figure 5:**
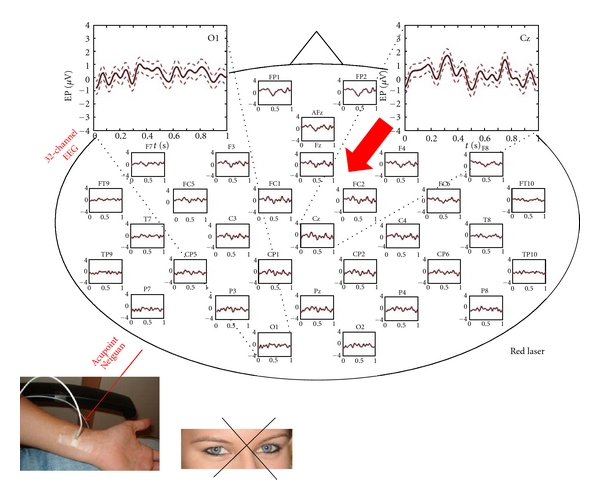
Reproducible evoked potentials after bilateral nonperceptible optical laser stimulation at the Neiguan acupuncture point. The evoked potentials are dominant over the central areas (red arrow) (*X*-axis: s; *Y*-axis: *μ*V; mean ± SE).

Figure 5 shows the same procedure; however the laser light stimulation at Neiguan acupuncture point was not visible and not perceptible for the subject (compare Figure 1). Only very small but reproducible evoked potentials are detectable, mainly over the central and frontal region.

**Figure 6:**
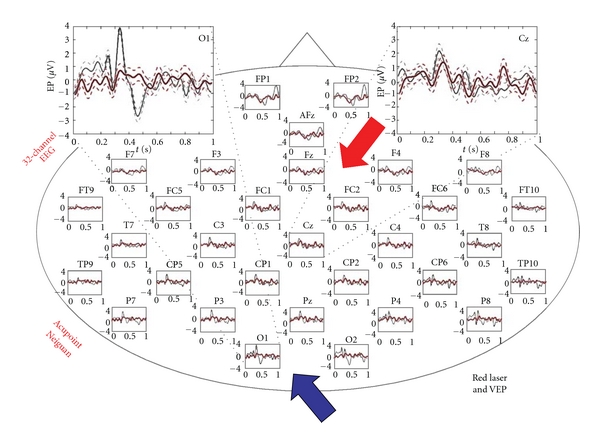
Comparison of visual and laser-induced evoked potentials plotted with the same scale (*X*-axis: s; *Y*-axis: *μ*V; mean ± SE).

To facilitate comparison between the results of the visual evoked potentials and the laser-induced evoked potentials, the two measurements are plotted on top of each other in Figure 6.

## 4. Discussion

An irradiation, as from a laser, is not a stimulus found in nature. Lasers (light amplification by stimulated emission of radiation) allow brief pulses (*μ*s to ms) with very fast rise time [18].

The laser needles used for acupuncture point stimulation in this study do not produce high temperatures; however, the penetration depth of the focused red laser light (658 nm) is about 3-4 cm [2]. Other experiments have indicated that brief high heating rate diode laser pulses can selectively activate myelinated A*δ* fiber nociceptors in rats and produce pricking pain in humans, whereas for low heating rate, longer pulses can preferentially activate unmyelinated C fibers in rats and produce burning pain in humans [18–22]. There is no evidence whether these different pulse parameters will differentially activate fibers in humans.

In our present study, the laser needle stimulation (658 nm) was not felt by the subject. It is very interesting that this nonperceptible optical stimulation can lead to cortical responses. This finding appears to be unique in literature. To the best of our knowledge, there are no studies in scientific literature describing this phenomenon. Some reports concerning the effects on human brain EEG caused by manual needle stimulation at the PC6 acupuncture site are available [17]. These authors found that the frequency peaks in alpha band of 12 channels were more synchronized during and after acupuncture [17].

Conscious perception of stimuli requires two intact systems. The first one is, the specific input system which results in an evoked potential. The second one is, the unspecific system called ARAS, the ascending reticular activating system which was first investigated by Moruzzi and Magoun [23].

There is an example which is similar to the mechanism probably at work in our experiment; for example, during sleep, the ear is also in an activated state; auditory evoked potentials are possible although one does not consciously perceive the stimuli. Therefore it could be possible that laser stimulation modulates functional structures in the ascending reticular activating system.

Further studies with EEG and other neuromonitoring techniques like near infrared spectroscopy [24] and different stimulation methods (optical-cave VEP, electrical, mechanical) are in progress and absolutely necessary to confirm or refute the preliminary findings.
